# Supplementary material for: The Systems Biology Research Tool: evolvable open-source software
Source: BMC Syst Biol. 2008 Jun 29;2:55. doi: 10.1186/1752-0509-2-55 (PMC2446383; doi:10.1186/1752-0509-2-55)
Supplement: Additional file 1 — SBRT Archive. An archive of the current version of the Systems Biology Research Tool. [file 1752-0509-2-55-S1.zip › sbrt-1.4.0/doc/developers_guide/api/sbrt/shell/io/TextFileLineIterator.html]

TextFileLineIterator


|  |  |  |
| --- | --- | --- |
| **Overview** \| **Package** \| **Class** \| **Use** \| **Tree** \| **Deprecated** \| **Index** \| **Help** | |  |
| **PREV CLASS**   **NEXT CLASS** | **FRAMES**    **NO FRAMES**     **All Classes** |  |
| SUMMARY: NESTED \| FIELD \| CONSTR \| METHOD | DETAIL: FIELD \| CONSTR \| METHOD |  |


---


## sbrt.shell.io Class TextFileLineIterator<E>

```
java.lang.Object
  sbrt.shell.io.FileLineIterator<E>
      sbrt.shell.io.TextFileLineIterator<E>
```

**Type Parameters:**: `E` - the returned type.

**All Implemented Interfaces:**: java.util.Iterator<E>

---

``` public final class TextFileLineIterator<E> extends FileLineIterator<E> ```

This class is used to iterate over the lines of an input file.

**Author:**
:   This class was written and documented by
    Jeremiah Wright while in the Wagner lab.

---

**Constructor Summary**

`TextFileLineIterator(TextInputFile<E> file)`
  
          Constructs a new text file line iterator using the
provided text input file.


| **Method Summary** | |
| --- | --- |
| `void` | `close()`             Closes this file iterator. |
| `protected  void` | `finalize()`             Disposes of system resources associated with this file iterator when called by the garbage collector. |
| `boolean` | `hasNext()`             Indicates if the file has more lines. |
| `boolean` | `isClosed()`             Indicates if this iterator has been closed. |
| `static boolean` | `isValidFormat(FileFormat fileFormat)`             Indicates if the provided file format is valid for this type of file line iterator. |
| `E` | `next()`             Returns the next element in the file. |
| `void` | `remove()`             Throws UnsupportedOperationException. |

**Methods inherited from class sbrt.shell.io.FileLineIterator**

`checkFile`

**Methods inherited from class java.lang.Object**

`clone, equals, getClass, hashCode, notify, notifyAll, toString, wait, wait, wait`

**Constructor Detail**

### TextFileLineIterator

```
public TextFileLineIterator(TextInputFile<E> file)
```

:   Constructs a new text file line iterator using the
    provided text input file.

    **Parameters:**: `file` - the file to be iterated over.


**Method Detail**

### isValidFormat

```
public static boolean isValidFormat(FileFormat fileFormat)
```

:   Indicates if the provided file format is valid for
    this type of file line iterator.

    :   **Parameters:**: `fileFormat` - the file format to test. **Returns:**: `true` if the provided file format is valid for a text input file; `false` otherwise.

---


### next

```
public E next()
```

:   Returns the next element in the file.

    :   **Returns:**: the next element in the file. **Throws:**: `java.util.NoSuchElementException` - if the file has no more elements.

---


### hasNext

```
public boolean hasNext()
```

:   Indicates if the file has more lines.

    :   **Returns:**: `true` if the file has more lines; `false` otherwise.

---


### remove

```
public void remove()
```

:   Throws UnsupportedOperationException.

    :   **Throws:**: `java.lang.UnsupportedOperationException`

---


### isClosed

```
public boolean isClosed()
```

:   Indicates if this iterator has been closed.

    :   **Returns:**: `true` if close() has been called; `false` otherwise.

---


### finalize

```
protected void finalize()
                 throws java.lang.Throwable
```

:   Disposes of system resources associated with this file
    iterator when called by the garbage collector.

    :   **Overrides:**: `finalize` in class `java.lang.Object`
    :   **Throws:**: `java.lang.Throwable` - the Exception raised by this method. **See Also:**: `Object.finalize()`

---


### close

```
public void close()
```

:   Closes this file iterator. If this file iterator is already
    closed, then invoking this method has no effect.

    :   **Throws:**: `ApplicationException` - if an I/O error occurred while closing this file iterator.


---


|  |  |  |
| --- | --- | --- |
| **Overview** \| **Package** \| **Class** \| **Use** \| **Tree** \| **Deprecated** \| **Index** \| **Help** | |  |
| **PREV CLASS**   **NEXT CLASS** | **FRAMES**    **NO FRAMES**     **All Classes** |  |
| SUMMARY: NESTED \| FIELD \| CONSTR \| METHOD | DETAIL: FIELD \| CONSTR \| METHOD |  |


---
